# Serum transthyretin and aminotransferases are associated with lean mass in people with coronary heart disease: Further insights from the CARE-CR study

**DOI:** 10.3389/fmed.2023.1094733

**Published:** 2023-02-20

**Authors:** Emily James, Stuart Goodall, Simon Nichols, Karen Walker, Sean Carroll, Alasdair F. O’Doherty, Lee Ingle

**Affiliations:** ^1^Department of Sport, Exercise and Rehabilitation, Northumbria University, Newcastle upon Tyne, United Kingdom; ^2^Diabetes Research Centre, University of Leicester, Leicester, United Kingdom; ^3^NIHR Leicester Biomedical Research Centre, Leicester, United Kingdom; ^4^Sport and Physical Activity Research Group, Sheffield Hallam University, Sheffield, United Kingdom; ^5^Advanced Wellbeing Research Centre, Sheffield Hallam University, Sheffield, United Kingdom; ^6^Department of Applied Sciences, Northumbria University, Newcastle upon Tyne, United Kingdom; ^7^School of Sport, Exercise and Rehabilitation Sciences, University of Hull, Hull, United Kingdom

**Keywords:** agrin, albumin, aminotransferases, biomarkers, coronary heart disease, muscle, sarcopenia, transthyretin

## Abstract

**Background:**

Low muscle mass disproportionately affects people with coronary heart disease compared to healthy controls but is under-researched and insufficiently treated. Inflammation, poor nutrition, and neural decline might contribute to low muscle mass. This study aimed to assess circulatory biomarkers related to these mechanisms [albumin, transthyretin, alanine aminotransferase (ALT), aspartate aminotransferase (AST), and C-terminal agrin fragment] and their relationship with muscle mass in people with coronary heart disease. Our findings could be beneficial to indicate mechanisms of sarcopenia, detect sarcopenia, and evaluate treatment.

**Methods:**

Serum blood samples from people with coronary heart disease were analysed for biomarker concentrations using enzyme-linked immunosorbent assays. Skeletal muscle mass was estimated using dual X-ray absorptiometry derived appendicular lean mass and reported as skeletal muscle index (SMI; kg m^−2^), and as a proportion of total body mass [appendicular skeletal mass (ASM%)]. Low muscle mass was defined as a SMI <7.0 and <6.0 kg m^−2^, or ASM% <25.72 and <19.43% for men and women, respectively. Associations between biomarkers and lean mass were adjusted for age and inflammation.

**Results:**

Sixty-four people were assessed; 14 (21.9%) had low muscle mass. People with low muscle mass had lower transthyretin (effect size 0.34, *p* = 0.007), ALT (effect size 0.34, *p* = 0.008), and AST (effect size 0.26, *p* = 0.037) concentrations, compared to those with normal muscle mass. SMI was associated with inflammation-corrected ALT (*r* = 0.261, *p* = 0.039) and with inflammation- and age-adjusted AST/ALT ratio (*r* = −0.257, *p* = 0.044). Albumin and C-terminal agrin fragment were not associated with muscle mass indices.

**Conclusion:**

Circulatory transthyretin, ALT and AST were associated with low muscle mass in people with coronary heart disease. Low concentrations of these biomarkers might indicate that low muscle mass is partially explained by poor nutrition and high inflammation in this cohort. Targeted treatments to address these factors could be considered for people with coronary heart disease.

## 1. Introduction

Between 1990 and 2019, coronary heart disease (CHD)-related mortality declined at a greater rate (61%) than CHD incidence (37%) (1). In the era of modern medical management, people with a CHD diagnosis live for longer and many will require increased support to manage their long-term health. An important component of healthy ageing is maintaining skeletal muscle mass (SMM) (2, 3). This is particularly relevant in people with CHD where there is a higher incidence of low SMM in people with CHD compared to age- and sex -matched adults (4). Emerging research in people with CHD shows that low SMM increases the risk of all-cause mortality, fatal or non-fatal major adverse cardiovascular events, lower fitness (peak oxygen uptake; V̇O_2peak_) and poorer quality of life (4–8). However, factors that influence loss of SMM in CHD are poorly defined. The delivery of successful interventions to improve SMM, and subsequently long-term health, in these people requires that we have: (1) the ability to identify those at risk of low SMM early, and (2) a thorough understanding of the factors influencing low SMM. For this purpose, circulatory biomarkers might be useful to complement traditional measures of SMM and strength.

Maladaptive processes and behaviours that contribute to loss of SMM and/ or function are complex. There is compelling evidence that these include neural maladaptation (9, 10), inflammation (11, 12), and sub-optimal nutrition (13, 14). Biomarkers which appear to have a central role in these systems need investigating. C-terminal agrin fragment (CAF) is a circulatory by-product of agrin cleavage by synaptic protease neurotrypsin (15), a process which can lead to neuromuscular junction breakdown (16). In healthy older adults (17, 18) and people with heart failure (19), CAF levels are elevated in those with low, compared to with normal, SMM. Thus, declining neural function might contribute to low SMM. However, it is unclear whether these findings exist in older people with CHD. Albumin and transthyretin are acute-phase response proteins which might indicate inflammation-related nutrition risk (20). In hospitalised people with CHD, albumin and transthyretin levels are lower in the presence of sarcopenia (as defined by the Asian Working Group for Sarcopenia) compared to those defined as non-sarcopenic (21). Whether albumin and transthyretin are associated with low SMM using European cut-off points (22), in people with CHD, requires clarification. Finally, alanine (ALT) and aspartate (AST) aminotransferases are liver/skeletal muscle enzymes (23). Circulatory levels of ALT are elevated in people with type 2 diabetes (24) and metabolic syndrome (25), but lower in the presence of age-related syndromes often characterised by under-nutrition, including sarcopenia (26). The AST/ALT ratio is proposed to be higher in those with sarcopenia compared to those without, although few studies have investigated this to date (27, 28).

Associations between SMM and serum CAF (17–19), albumin, transthyretin (21), ALT and AST (26–28) were reported in healthy older adults and people with chronic health conditions. The present study aimed to investigate the association between estimated SMM, and serum CAF, albumin, transthyretin, ALT and AST, in people with recently diagnosed stable CHD. We hypothesised that people with CHD and low SMM will have higher CAF levels and AST/ALT ratio and lower albumin and transthyretin levels, compared to people with CHD and preserved SMM.

## 2. Materials and methods

### 2.1. Study design and participants

Baseline serum blood samples and demographic characteristics used in this cross-sectional study were collected as part of the Cardiovascular and cardiorespiratory Adaptations to Routine Exercise-based Cardiac Rehabilitation (CARE CR) study (29). The CARE CR study protocol was published in detail elsewhere (29). Briefly, clinically stable people with a primary diagnosis of CHD (aged 30–85 years) were referred to the research team by nursing staff, within 2 weeks of a cardiac event or procedure. Participants provided their written informed consent to participate in the study. The CARE CR study was granted ethical approval by the Humber Bridge NHS Research Ethics Committee-Yorkshire and the Humber (12/YH/0278). Ethical approval for assay analysis of serum samples for biomarkers related to sarcopenia was provided by the Northumbria University Health and Life Sciences Ethics Committee (20933). The main findings from the CARE CR study on patient rehabilitation and cardiorespiratory fitness are published elsewhere (4, 30).

### 2.2. Body composition

Body mass index (BMI; kg m^−2^) was calculated using mass (kg) and stature (m). Waist and hip circumferences (cm) were measured at 1 cm above the iliac crest and at the widest aspect of the hips, respectively. Appendicular lean mass (ALM), defined as total lean mass in both arms and legs (kg), was measured using dual X-ray absorptiometry (DXA; Lunar iDXA GE Healthcare Buckinghamshire, United Kingdom), as a proxy for SMM assessment. ALM is expressed as skeletal muscle index (SMI; kg m^−2^) and as a percentage of total body mass (appendicular skeletal mass; ASM%). Age-adjusted SMI and ASM% were moderately correlated (*r* = 0.507, *p* < 0.001). We defined low SMI as <7.0 and <6.0 kg m^−2^ (22) and low ASM% as <25.72 and <19.43% (31) for men and women, respectively.

### 2.3. Maximal cardiopulmonary exercise test

Cardiopulmonary exercise testing was performed using the modified Bruce treadmill protocol (32), as previously described (4, 29). A 12-lead Electrocardiogram (ECG), ECG-gated automated blood pressure, heart rate, and rate of perceived exertion were monitored throughout. Breath-by-breath metabolic gas exchange data were collected using an Oxycon Pro metabolic cart (Jaeger, Hoechburg, Germany). We report V̇O_2peak_ (ml), defined as the mean V̇O_2_ over the last 30 s of the test; V̇O_2peak_ was adjusted for body mass (ml kg^−1^ min^−1^) (4).

### 2.4. Blood sampling and analysis

Participants abstained from strenuous exercise 24-h prior to attending their baseline study visit. Resting blood samples were drawn by venepuncture and placed in a refrigerated (4°C) centrifuge at 3,000 revolutions per minute, for 15 min. Albumin, aminotransferases and N-terminal pro-brain natriuretic peptide (NT-proBNP) were analysed at the Hull Royal Infirmary in an accredited biochemistry laboratory, as a single measurement on the day of each blood draw. Calibration and quality controls were conducted in accordance with manufacturer’s guidelines. The ABX Pentra 400 biochemistry auto analyser (Horiba, Montpellier, France) was used to analyse high sensitivity C-reactive protein (hs-CRP) in duplicate, in accordance with the manufacturer’s quality control guidance (4). Remaining plasma and serum samples were stored at −80°C until analysis.

We analysed serum samples in duplicate using commercial enzyme-linked immunosorbent assay (ELISA) for CAF (Abcam #ab216945) and transthyretin (Abcam #ab108895) and followed their standard instructions for serum analysis. Concentrations of transthyretin and CAF were assessed in duplicate and the average of the two measures reported. We re-analysed samples with a coefficient of variation (CV) >40% and when biomarker concentrations were not within the limits of the standard curve. The CV for the assay analyses of transthyretin and CAF were 7.9 and 5.1%, respectively. Routine health-related serum biomarkers evaluated as part of the CARE CR study are reported elsewhere, including NT-proBNP, hs-CRP, glucose, white cell count, total cholesterol, low-density and high-density lipoprotein cholesterol, estimated glomerular filtration rate and triglycerides (4, 30).

Normal adult reference values for circulatory markers of interest are:

- Albumin: 35–50 g/L (33).
- Transthyretin: 30–33 and 25–27 mg/dl in males and females, respectively (34).
- ALT: 9.0–59.0 and 7.8–41.0 U/L in males and females, respectively (35).
- AST: 11.0–34.0 U/L (35)
- CAF: 0.86–4.66 ng/ml (17).

### 2.5. Statistical analysis

Statistical analyses were performed by a single researcher using commercially available software (SPSS version 28, IBM, New York, NY, United States). Distribution of the data was assessed using visual inspection of histograms, QQ-plots and using the Kolmogorov Smirnov test. Categorical variables are reported as frequency with percentage. Continuous normally distributed variables are reported as mean ± standard deviation. Continuous non-normally distributed variables are reported as median with interquartile range, or median with range where the sample size is ≤3 people. Demographic characteristics are reported for the whole cohort and separately for people with normal or low SMM (defined as low SMI or low ASM%). Differences in demographic characteristics between the two groups were assessed using the Fisher’s exact test (categorical variables), a Student’s *t*-test (continuous normally distributed), or Mann–Whitney *U* test (continuous non-normally distributed). Two-group comparison of blood biomarkers between people with normal or low SMM were evaluated using Mann–Whitney *U* tests and reported as U statistics, *p*-values, and effect sizes, calculated using the following equation (36):


$$r=\frac{Z}{\surd n}$$

Absolute r values of 0.2, 0.5, and 0.8 are considered small, moderate and large effect sizes, respectively (37). The relationship between serum biomarker concentrations, SMI and ASM% were calculated using Spearman’s rank correlations. It is well-established that age and inflammation influence SMM and some serum biomarkers; people with CHD and low SMM are significantly older than those with normal SMM (38), whilst albumin and transthyretin concentrations decrease in the presence of inflammation (39). Accordingly, we also report non-parametric partial correlations adjusted for age and circulatory hs-CRP concentrations, both separately and together. An r value of <0.3, 0.3–0.5, 0.6–0.8, and >0.8 indicated a poor, fair, moderately strong and very strong associations, respectively (40). Scatterplots of associations between SMI and circulatory markers were plotted with linear regression lines. Where a marker was associated with SMI or ASM% or had a significant effect size for low and normal SMM groups, receiver operating characteristic (ROC) curves were used to investigate the sensitivity and specificity of predicting low SMM as the dichotomous ‘state variable’. We report the area under the curve (AUC) with 95% confidence interval (CI) and *p*-values. The AUC value was interpreted as follows: perfect (1.0), excellent (0.9–0.99), good (0.8–0.89), fair (0.7–0.79), poor (0.51–0.69), and no value (0.5) (41). The biomarker concentration cut-off points for prediction of low SMM were selected based on the highest combination of sensitivity and specificity values. We plotted ROC curves and determined biomarker cut-off points for the whole cohort and then separately for men only. Due to a small sample, ROC curves could not be plotted for women only. Statistical significance was set at *p* < 0.05.

## 3. Results

Sixty-four people were included (63.4 ± 9.8 years; 12.5% female). Participant characteristics, presenting diagnosis, comorbidities, and medications, are reported in Table 1. Low ASM% and low SMI were identified in 14.1% (*n* = 9) and 12.5% (*n* = 8) of people, respectively. Three people had both low ASM% and low SMI (4.7%) and 14 had either low ASM% or low SMI (21.9%).

**Table 1:** Patient baseline characteristics.

Variable	Mean ± standard deviation or frequency (%)		
	All people (*n* = 64)	Low SMM (*n* = 14)	Normal SMM (*n* = 50)
Age (years)	63.4 ± 9.8	67.6 ± 10.6	62.2 ± 9.4
Female	8 (12.5)	5 (35.7)*	3 (6.0)
Body mass index (kg m^−2^)	28.9 ± 3.9	28.9 ± 5.5	28.9 ± 3.3
Body fat content (%)	36.1 ± 6.9	41.9 ± 8.6**	34.5 ± 5.4
Waist/ Hip circumferences ratio^a^^,^^b^	0.97 (0.93, 1.02)	0.96 (0.86, 1.0)	0.97 (0.93, 1.0)
Appendicular lean mass (kg)	23.8 ± 4.6	18.9 ± 3.2**	25.2 ± 3.9
Skeletal muscle index (kg m^−2^)	8.7 ± 1.7	6.9 ± 1.2**	9.2 ± 1.4
Appendicular skeletal mass (%)^a^	28.7 (26.2, 30.8)	24.4 (22.0, 25.3)**	29.1 (27.9, 31.0)
V̇̇O_2peak_ (ml kg^−1^ min^−1^)	23.9 ± 6.0	20.1 ± 5.6*	24.6 ± 5.8
Left ventricular ejection fraction (%)	55.1 ± 7.0	53.9 ± 8.7	55.5 ± 6.5
*N*-terminal pro-brain natriuretic peptide (NT-proBNP; pg L^−1^)^a^^,^^b^	172.0 (64.7, 344.0)	357.0 (112.8, 998.0)*	138.0 (55.8, 273.5)
**Presenting diagnosis**			
ST-elevation MI (STEMI)	14 (21.9)	3 (21.4)	11 (22.0)
Non-ST-elevation MI (non-STEMI)	21 (32.8)	4 (28.6)	17 (34.0)
Elective percutaneous coronary intervention (PCI)	17 (26.6)	3 (21.4)	14 (28.0)
Coronary artery bypass graft (CABG)	6 (9.4)	2 (14.3)	4 (8.0)
Angina	6 (9.4)	2 (14.3)	4 (8.0)
**Comorbidities**			
Hypertension	30 (46.9)	8 (57.1)	22 (44.0)
Type 2 diabetes	12 (18.8)	3 (21.4)	9 (18.0)
Chronic obstructive pulmonary disease (COPD)	3 (4.7)	2 (14.3)	1 (2.0)
Hyperlipidaemia	43 (67.2)	9 (64.3)	34 (68.0)
Previous PCI	13 (20.3)	4 (28.5)	9 (18.0)
Previous MI	13 (20.3)	2 (14.2)	11 (22.0)
Previous CABG	5 (7.8)	2 (14.3)	3 (6.0)
Previous cardiac valve surgery	1 (1.6)	0	1 (2.0)
Previous transient ischemic attack	6 (9.4)	3 (21.4)	3 (6.0)
Cancer	10 (15.6)	5 (35.7)*	5 (10.0)
**Medications**			
Aspirin	62 (96.9)	13 (92.9)	49 (98.0)
Clopidogrel	19 (29.7)	6 (42.9)	13 (26.0)
Ticagrelor	32 (50.0)	4 (28.6)	28 (56.0)
Beta-blockers	57 (89.1)	12 (85.7)	45 (90.0)
Angiotensin converting enzyme (ACE)-inhibitors	38 (59.4)	10 (71.4)	28 (56.0)
Statins	61 (95.3)	14 (100.0)	47 (94.0)
Diuretics	7 (10.9)	3 (21.4)	4 (8.0)
Nitrates (non-GTN)	15 (23.4)	2 (14.3)	13 (26.0)
GTN spray	58 (90.6)	12 (85.7)	46 (92.0)

CABG, coronnary artery bypass graft; GTN, glyceryl trinitrate; MI, myocardial infarction. *p < 0.05 or **p < 0.01 compared to normal SMM group.

a Values are median (interquartile range).

b *n* = 63.

Circulatory biomarker concentrations are reported in Table 2. The distribution of biomarker concentrations compared to normal reference values (section 2.4) were as follows: albumin, 92.2% (*n* = 59) within, 6.3% (*n* = 4) lower than and 1.6% (*n* = 1) higher than the normal range; transthyretin, 6.3% (*n* = 4) within, 34.4% (*n* = 22) lower than and 59.4% (*n* = 38) higher than the normal range; ALT, 90.6% (*n* = 58) within and 9.4% (*n* = 6) higher than the normal range; AST, 78.1% (*n* = 50) within and 21.9% (*n* = 14) higher than the normal range; and CAF, 78.1% (*n* = 50) within and 21.9% (*n* = 14) higher than the normal range. There were small to moderate effect sizes for lower serum transthyretin (effect size 0.34; 29.66 mg/dl versus 37.87 mg/dl, *p* = 0.007), ALT (effect size 0.34; 20.00 U/L versus 31.00 U/L, *p* = 0.008) and AST (effect size 0.26; 22.25 U/L versus 27.00 U/L, *p* = 0.037) levels in people with low SMM compared to those with normal SMM.

**Table 2:** Circulatory biomarker concentrations in people with coronary heart disease with low or normal skeletal muscle mass (SMM).

Biomarker	All					Men					Women				
	Low SMM (*n* = 14)	Normal SMM (*n* = 50)	U	ES	*p*-Value	Low SMM (*n* = 9)	Normal SMM (*n* = 47)	U	ES	*p*-Value	Low SMM (*n* = 5)	Normal SMM (*n* = 3)	U	ES	*p*-Value
Albumin (g/L)	37.50 (36.00, 39.25)	38.50 (37.00, 41.00)	283.00	0.14	0.274	38.00 (36.00, 39.50)	39.00 (37.00, 41.00)	170.00	0.12	0.352	37.00 (36.00, 40.00)	37.00 (36.00, 38.00)	7.00	0.05	0.877
Transthyretin (mg/dl)	29.66 (18.36, 34.08)	37.87 (28.83, 53.63)	183.00	0.34	0.007**	28.64 (17.51, 34.96)	37.88 (28.87, 54.55)	96.00	0.34	0.010*	32.07 (22.23, 35.28)	24.34 (24.08, 50.34)	7.00	0.05	0.881
Alanine aminotransferase (U/L)	20.00 (17.00, 24.00)	31.00 (21.75, 41.25)	188.00	0.34	0.008**	20.00 (19.50, 24.00)	32.00 (22.00, 42.00)	127.50	0.25	0.061	17.00 (14.50, 28.00)	21.00 (19.00, 24.00)	3.00	0.47	0.180
Aspartate aminotransferase (U/L)	22.25 (18.00, 29.13)	27.00 (23.00, 34.75)	221.50	0.26	0.037*	24.00 (22.25, 31.50)	27.50 (23.00, 35.50)	186.00	0.08	0.569	18.00 (17.25, 20.25)	25.50 (22.50, 27.00)	0.00	0.79	0.025*
AST/ALT	1.17 (0.93, 1.25)	0.91 (0.69, 1.23)	255.00	0.19	0.123	1.20 (0.92, 1.31)	0.86 (0.68, 1.23)	130.00	0.25	0.069	1.13 (0.76, 1.24)	1.07 (1.06, 1.42)	6.00	0.16	0.655
C-terminal agrin fragment (ng/ml)	3.89 (3.10, 4.24)	3.67 (3.11, 4.48)	335.00	0.03	0.808	4.15 (2.55, 4.53)	3.63 (3.05, 4.36)	206.00	0.02	0.902	3.74 (3.37, 4.02)	4.83 (4.02, 5.25)	1.00	0.69	0.053
Hs C-reactive protein (mg/L)^a^	2.51 (0.42, 4.33)	1.19 (0.50, 3.41)	303.00	0.10	0.445	2.62 (0.58, 4.63)	1.18 (0.48, 3.41)	164.00	0.14	0.289	1.87 (0.32, 4.25)	2.96 (2.17, 8.86)	4.00	0.37	0.297

Bold values indicate statistical significance (*p* < 0.05). *p < 0.05 or **p < 0.01 compared to normal SMM group. AST/ALT, alanine aminotransferase/aspartate aminotransferase ratio; ES, effect size; U, U statistic from Mann–Whitney *U* test. Low SMM was skeletal muscle index <7.0 and <6.0 kg m^−2^, or appendicular skeletal muscle <25.72 and <19.43%, for men and women, respectively. Values are median (range) where *n* = 3. All other values are median (interquartile range).

a Values from a subset of people included in the present study were been reported elsewhere (4).

**Figure 1:**
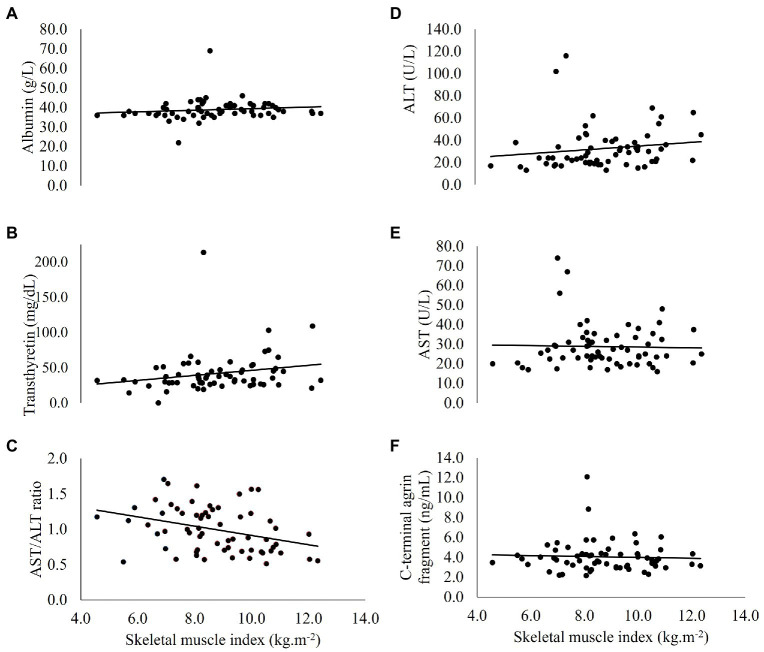
Correlations between skeletal muscle index and circulatory **(A)** albumin, **(B)** transthyretin, **(C)** AST/ALT ratio, **(D)** ALT, **(E)** AST, and **(F)** C-terminal agrin fragment, in people with coronary heart disease (*n* = 64). ALT, alanine aminotransferase; AST, aspartate aminotransferase.

Correlations between circulatory biomarkers, SMI and ASM% are reported in Table 3. Figure 1 shows correlations between SMI and circulatory biomarkers. SMI was associated with hs-CRP -corrected serum ALT levels (*r* = 0.261, *p* = 0.039) and with hs-CRP and age -corrected AST/ALT ratio (*r* = −0.257, *p* = 0.044). In men, after correction for hs-CRP levels and age, SMI was associated with AST (*r* = −0.279, *p* = 0.041) and the AST/ALT ratio (*r* = −0.281, *p* = 0.040). In women, after correction for hs-CRP levels and age, transthyretin was negatively associated with ASM% (*r* = −0.889, *p* = 0.018).

**Table 3:** Correlations between SMI, ASM%, and serum biomarkers.

	All (*n* = 64)		Men (*n* = 56)		Women (*n* = 8)	
	SMI	ASM%	SMI	ASM%	SMI	ASM%
*Albumin*						
Spearman’s corr. (*r*)	0.229	0.179	0.147	0.104	0.593	0.272
*p*-value	0.069	0.157	0.279	0.447	0.121	0.515
Partial corr. (*r*)^a^	0.217	0.141	0.143	0.071	0.738	0.114
*p*-value	0.087	0.271	0.297	0.607	0.058	0.807
Partial corr. (*r*)^b^	0.082	0.151	−0.023	0.081	0.765*	0.198
*p*-value	0.524	0.236	0.868	0.557	**0.045**	0.671
Partial corr. (*r*)^c^	0.072	0.116	−0.034	0.036	0.763	0.248
*p*-value	0.579	0.369	0.809	0.795	0.078	0.636
*Transthyretin*						
Spearman’s corr. (*r*)	0.246	0.132	0.208	0.048	0.048	−0.857**
*p*-value	**0.050**	0.297	0.124	0.725	0.911	**0.007**
Partial corr. (*r*)^a^	0.237	0.101	0.204	0.002	0.030	−0.839*
*p*-value	0.061	0.433	0.135	0.987	0.949	**0.018**
Partial corr. (*r*)^b^	0.213	0.120	0.142	0.033	0.075	−0.874
*p*-value	0.094	0.349	0.302	0.808	0.872	**0.010** *
Partial corr. (*r*)^c^	0.206	0.090	0.134	−0.017	0.054	−0.889*
*p*-value	0.109	0.484	0.335	0.901	0.919	**0.018**
*Alanine aminotransferase (ALT)*						
Spearman’s corr. (*r*)	0.271*	0.209	0.144	0.048	0.190	0.095
*p*-value	**0.030**	0.098	0.289	0.726	0.651	0.823
Partial corr. (*r*)^a^	0.261*	0.175	0.141	0.023	0.200	0.051
*p*-value	**0.039**	0.170	0.304	0.868	0.668	0.913
Partial corr. (*r*)^b^	0.158	0.186	−0.032	0.016	0.193	0.106
*p*-value	0.216	0.144	0.819	0.917	0.678	0.820
Partial corr. (*r*)^c^	0.150	0.156	−0.040	−0.020	0.277	−0.016
*p*-value	0.245	0.226	0.776	0.886	0.596	0.977
*Aspartate aminotransferase (AST)*						
Spearman’s corr. (*r*)	0.038	0.181	−0.169	−0.001	0.238	0.190
*p*-value	0.766	0.152	0.213	0.993	0.570	0.651
Partial corr. (*r*)^a^	0.034	0.176	−0.171	−0.013	0.236	0.339
*p*-value	0.791	0.168	0.212	0.927	0.610	0.457
Partial corr. (*r*)^b^	−0.017	0.168	−0.275*	−0.018	0.277	0.290
*p*-value	0.897	0.188	**0.042**	0.895	0.547	0.528
Partial corr. (*r*)^c^	−0.019	0.165	−0.279*	−0.033	0.279	0.313
*p*-value	0.825	0.199	**0.041**	0.812	0.592	0.546
*AST/ALT ratio*						
Spearman’s corr. (*r*)	−0.360**	−0.089	−0.386**	−0.031	−0.048	0.000
*p*-value	**0.003**	0.484	**0.003**	0.823	0.911	1.00
Partial corr. (*r*)^a^	−0.351**	−0.043	−0.384**	−0.001	−0.117	0.361
*p*-value	**0.005**	0.736	**0.004**	0.997	0.803	0.427
Partial corr. (*r*)^b^	−0.264*	−0.054	−0.285*	−0.002	−0.025	0.135
*p*-value	**0.036**	0.675	**0.035**	0.986	0.958	0.773
Partial corr. (*r*)^c^	−0.257*	−0.011	−0.281*	0.037	−0.139	0.393
*p*-value	**0.044**	0.932	**0.040**	0.792	0.793	0.441
*C-terminal agrin fragment*						
Spearman’s corr. (*r*)	−0.042	−0.180	−0.008	−0.161	0.429	0.286
*p*-value	0.741	0.154	0.956	0.235	0.289	0.493
Partial corr. (*r*)^a^	−0.029	−0.146	−0.002	−0.133	0.429	0.303
*p*-value	0.823	0.253	0.988	0.334	0.337	0.509
Partial corr. (*r*)^b^	−0.048	−0.182	−0.015	−0.163	0.436	0.311
*p*-value	0.706	0.152	0.912	0.234	0.329	0.497
Partial corr. (*r*)^c^	−0.038	−0.148	−0.008	−0.134	0.476	0.277
*p*-value	0.770	0.250	0.955	0.333	0.340	0.595

Bold values indicate statistical significance (*p* < 0.05). corr., correlation; ASM%, appendicular skeletal muscle; SMI, skeletal muscle index (kg m^−2^). Non-parametric partial correlations are corrected for (a) high-sensitivity C-reactive protein, (b) age, and (c) high-sensitivity C-reactive protein and age.

* *p* < 0.05;

** *p* < 0.01.

### 3.1. ROC curve analysis

The prognostic value of transthyretin, ALT, AST, and the AST/ALT ratio for identification of low SMM was assessed using ROC curve analysis. Including all participants, transthyretin (AUC 0.739, 95% CI 0.601, 0.876, *p* = 0.007) and ALT (AUC 0.731, 95% CI 0.576, 0.887, *p* = 0.009) had the greatest predictive capacity to identify low SMM. The AUC for AST level was 0.684 (95% CI 0.516, 0.851, *p* = 0.037) and non-significant for the AST/ALT ratio (AUC 0.636, 95% CI 0.482, 0.790, *p* = 0.123). The optimal cut-off points to indicate risk of low SMM were: a transthyretin value of ≤37.7654 mg/dl (sensitivity 0.857, specificity 0.520), an ALT value of ≤25.00 U/L (sensitivity 0.857, specificity 0.620), and an AST value of ≤24.50 U/L (sensitivity 0.714, specificity 0.620).

Including men only, ROC curve analyses showed the predictive capacity of transthyretin (AUC 0.773, 95% CI 0.603, 0.943, *p* = 0.002), ALT (AUC 0.699, 95% CI 0.509, 0.888, *p* = 0.040) and the AST/ALT ratio (AUC 0.693, 95% CI 0.538, 0.847, *p* = 0.014). The AUC for AST level was non-significant (AUC 0.560, 95% CI 0.357, 0.764, *p* = 0.562). In men, the optimal cut-off points to indicate risk of low SMM where: a transthyretin value of ≤30.3284 mg/dl (sensitivity 0.778, specificity 0.723), an ALT value of ≤25.00 U/L (sensitivity 0.889, specificity 0.660), and an AST/ALT ratio of ≥0.9347 (sensitivity 0.778, specificity 0.553).

## 4. Discussion

This study aimed to report the association between DXA-estimated SMM and serum albumin, transthyretin, ALT, AST, and CAF in people with CHD. People with low SMM had lower serum transthyretin, AST and ALT levels compared to those with normal SMM, with small to moderate effect sizes. SMI was positively associated with ALT level and negatively associated with the AST/ALT ratio. We found no associations between albumin or CAF levels with any SMM index.

More than one-fifth of people had low SMM. Similarly, others report a prevalence of 25–30% for low SMM in people with CHD (5, 7, 42). Comparatively fewer (12%) apparently healthy, community-dwelling, older adults have low SMM (43). In the current study, presence of comorbidities associated with SMM loss, such as cancer (44) and COPD (45), likely contributed to the higher prevalence of low SMM. Importantly, in a previous CARE CR publication, ASM% was inversely associated with estimated all-cause mortality risk (*r* = −0.365, *p* = 0.006) in people with CHD (4). Thus, interventions to prevent or reverse low SMM should be offered to these people. To support the design and implementation of successful interventions, accurate and readily available methods to assess or monitor changes in SMM are needed.

### 4.1. Albumin

Albumin is a marker of inflammation-related nutritional risk (20). In agreement with previous studies involving people with liver cirrhosis (46), end-stage renal disease (47) and heart failure (48), we found no association between albumin levels and SMM indices in people with CHD. Interestingly, others report both lower (49–51) and or higher (52) albumin concentrations in older adults with low SMM, compared to those with preserved SMM. The use of albumin levels to infer protein energy malnutrition was previously commonplace in clinical practise (53). Given that lean mass reflects the somatic protein store, the assumption followed that albumin might be useful as a marker of lean mass. However, the use of albumin as a biomarker of malnutrition or body composition has not been without criticism (20, 54). The literature lacks consensus on the existence and/ or direction of the association between albumin and SMM-related variables (46–49, 51, 52), likely due to the role of albumin as an acute-phase response protein.

The inflammation-induced reduction in albumin concentration is underpinned by: decreased albumin synthesis during stress response to prioritise synthesis of essential proteins, increased capillary permeability prompting a shift of albumin from the intravascular to the interstitial space, and a shortened albumin half-life resulting from tissue catabolism (20). In older adults, serum albumin is inversely associated with common inflammatory cytokine, CRP (55). We found no difference in hs-CRP between people with normal or low SMM (Table 2). This could explain the similar albumin levels between groups. Additionally, Chen et al. (56) speculated that sex-specific hormones levels might also impact the association between SMM and albumin levels, after finding these variables to be positively associated in men and negatively associated in women. However, our study included a small sample of women, and we were unable to investigate this hypothesis.

### 4.2. Transthyretin

Transthyretin levels were significantly lower in people with low versus normal SMM. Similar to albumin, transthyretin is a marker of inflammation-related nutritional risk (20), a key component of malnutrition related to acute or chronic disease (57). Amino acid availability, from dietary protein intake, was proposed to mediate the relationship between transthyretin and lean mass (34). This is because amino acid ingestion promotes lean tissue accretion (58) and also modulates transthyretin synthesis in the liver (59). A strong, positive association (*r* = 0.58) between transthyretin levels and SMI was previously reported in people at a geriatric outpatient hospital (51). Around 40% of people in the study by Sergi et al. (51) were underweight (BMI <20 kg m^−2^). Poorer nutritional status likely contributed to a more pronounced inflammatory environment and lean mass loss in this study (51), potentially explaining the strong association between transthyretin and SMI, compared to a non-significant association in the present study (*r* = 0.246, *p* = 0.05). Nevertheless, our detection of significantly lower transthyretin levels in people with low compared to normal SMM is a promising finding, as it become increasingly apparent that transthyretin assessment might have clinical utility as part of a comprehensive medical evaluation (60).

### 4.3. Aminotransferases

Assessment of liver enzymes ALT and AST is routine in clinical practise (61). As a catalyst in the alanine-glucose cycle, ALT converts pyruvate to amino acid alanine in skeletal muscle and converts alanine back to pyruvate (for glucose production) in the liver (62, 63). A similar cycle is catalysed by AST, where the amino acid and product are aspartate and oxaloacetate, respectively (63). Circulatory levels of ALT and AST are elevated in Type 2 diabetes (24) and metabolic syndrome (25), conditions characterised by insulin resistance and hepatic steatosis. We, and others, demonstrate that ALT levels appear to be lower in the presence of low SMM (26, 50). Contrastingly, in a cross-section of >12,000 adults without liver-related disorders, ALT levels were elevated in those with low SMM compared to normal SMM (64). The direction of the relationship between AST and SMM is similarly contested. We found lower AST concentrations in people with low SMM compared to normal SMM. Others report that low SMM coincided with higher AST concentrations in people with (65, 66) and without (64) liver disease.

Multiple factors likely influence the inconsistency in these findings. First, damaged liver cells release ALT and AST into circulation, explaining their higher serum concentrations in people with liver disorders (67). Secondly, participants with low SMM in the study by Yoo et al. (64) were more often obese with higher fasting blood glucose and insulin levels compared to the normal SMM group, consistent with the theory that aminotransferase levels are elevated in the presence of higher metabolic risk. In the present study, people with reduced SMM had higher average body fat and comparable BMI to people with normal SMM. It could be speculated that differences in intra-abdominal and intra-hepatic steatosis, together with diet quality/alcohol consumption might have influenced aminotransferase concentrations.

Additionally, both ALT and AST require vitamin B_6_ as a cofactor, meaning that vitamin B_6_ deficiency might contribute to low circulatory ALT and AST (68). Furthermore, vitamin B_6_ is mostly stored in striated muscle (69); thus, where lean mass is reduced a smaller pool of vitamin B_6_ is available to act as a cofactor for AST and ALT. An estimated 31 and 24% of community-dwelling men and women (≥65 years) are at risk of inadequate vitamin B_6_ dietary intake (70). Although not assessed in this study, addressing any dietary deficiencies in people with CHD and low SMM should be prioritised.

### 4.4. C-terminal agrin fragment

Studies involving older adults (17, 71, 72), people with lung disease (72, 73) and with heart failure (19, 73) have reported an association between high circulatory CAF levels and low SMM. This association is proposed to originate from degeneration of the neuromuscular junction with ageing. Agrin is cleaved by neurotrypsin during normal neural development (15). Excessive agrin cleavage from over-expression of neurotrypsin causes agrin to become deactivated and the neuromuscular junction to break down (16). The product of this breakdown, CAF, is released into the circulation (74). However, the effect of degeneration and remodelling of the neuromuscular junction on SMM loss is debated, with polarising studies arguing that this process contributes to (75) or is protective against (76) muscle atrophy.

We found no association between CAF levels and SMM indices in people with CHD. Others have reported similar non-significant findings when assessing possible associations between CAF and presence of frailty in people with CHD, although an assessment of SMM was not included in their definition of frailty (77). Sánchez-Castellano et al. (78) found no difference in CAF levels between low and normal SMM groups with hip fracture and suggested that elevated CAF levels in both groups indicated neuromuscular degeneration was present in both. In contrast, median CAF values in the low and normal SMM groups were within the normal limits in the present study (0.86–4.66 ng/ml; 17), suggesting that circulatory CAF has limited utility as biomarker for low SMM in this cohort.

### 4.5. Strengths and limitations

We assessed, in a secondary analysis, multiple proposed biomarkers for low SMM in people with CHD, contributing to our understanding of the factors influencing this complex and under-researched pathology. We included assessment of four biomarkers which are already commonly assessed in clinical practise (albumin, transthyretin, ALT, and AST), aiding the potential transition of our findings into practise.

This study is potentially limited by our use of DXA-derived lean mass to estimate SMM. DXA assessment is the current reference standard, but is limited by the production of variability related to different devices and software versions (79) and the absence of a universally agreed cut-off point for low SMM (80). Furthermore, DXA derived lean mass can be interpreted in several ways (i.e., corrected for stature, body mass or body fat percentage), which often produce conflicting findings when analysed in relation to circulatory biomarkers. This might limit the comparability of our findings with other, similar research. Finally, we included a small sample of women and there was no assessment of muscle strength or function.

### 4.6. Future research

Future research should evaluate the association between albumin, transthyretin, aminotransferases, CAF and measures of muscular strength alongside SMM. Whether these markers change with targeted lifestyle interventions also requires investigation. Additionally, there appears to be sex differences in median biomarker concentrations and their correlations with SMM indices, although our small sample of women limits the certainty of this finding. Future research might further investigate sex differences in SMM biomarkers in people with CHD.

## 5. Conclusion

This study aimed to identify associations between SMM indices and circulatory biomarkers in people with CHD. Lower levels of serum transthyretin, AST and ALT were present in people with CHD and low SMM, compared to those with normal SMM. To assist with practical application, we also identified the cut-off points below which transthyretin, ALT and AST indicate high likelihood of low SMM. We found no association between albumin, CAF and SMM indices, suggesting that these markers have limited utility as markers for low SMM in this cohort.

## Data availability statement

The raw data supporting the conclusions of this article will be made available by the authors, without undue reservation.

## Ethics statement

The studies involving human participants were reviewed and approved by Humber Bridge NHS Research Ethics Committee- Yorkshire and the Humber and Northumbria University Health and Life Sciences Ethics Committee. The patients/participants provided their written informed consent to participate in this study.

## Author contributions

AO’D, EJ, SN, LI, SG, and SC: conceptualization. SN, AO’D, EJ, and KW: data collection. EJ: analysis and writing original draft. All authors contributed to the article and approved the submitted version.

## Funding

Financial support for blood sample analysis was provided by Hull and East Riding Cardiac Trust Fund (Hull, East Yorkshire, United Kingdom) and Northumbria University (Newcastle upon Tyne, United Kingdom).

## Conflict of interest

The authors declare that the research was conducted in the absence of any commercial or financial relationships that could be construed as a potential conflict of interest.

## Publisher’s note

All claims expressed in this article are solely those of the authors and do not necessarily represent those of their affiliated organizations, or those of the publisher, the editors and the reviewers. Any product that may be evaluated in this article, or claim that may be made by its manufacturer, is not guaranteed or endorsed by the publisher.

## Abbreviations

ALT: Alanine aminotransferase
ASM%: Appendicular skeletal muscle
AST: Aspartate aminotransferase
CAF: C-terminal agrin fragment
CHD: Coronary heart disease
DXA: Dual X-ray absorptiometry
Hs-CRP: High sensitivity C-reactive protein
SMI: Skeletal muscle index
SMM: Skeletal muscle mass
